# Extent of Spinal Cord Decompression in Motor Complete (American Spinal Injury Association Impairment Scale Grades A and B) Traumatic Spinal Cord Injury Patients: Post-Operative Magnetic Resonance Imaging Analysis of Standard Operative Approaches

**DOI:** 10.1089/neu.2018.5834

**Published:** 2019-02-27

**Authors:** Bizhan Aarabi, Joshua Olexa, Timothy Chryssikos, Samuel M. Galvagno, David S. Hersh, Aaron Wessell, Charles Sansur, Gary Schwartzbauer, Kenneth Crandall, Kathirkamanathan Shanmuganathan, J. Marc Simard, Harry Mushlin, Mathew Kole, Elizabeth Le, Nathan Pratt, Gregory Cannarsa, Cara D. Lomangino, Maureen Scarboro, Carla Aresco, Brian Curry

**Affiliations:** ^1^ Department of Neurosurgery, University of Maryland School of Medicine, Baltimore, Maryland.; ^2^ Department of Anesthesiology, University of Maryland School of Medicine, Baltimore, Maryland.; ^3^ R. Adams Cowley Shock Trauma Center, University of Maryland School of Medicine, Baltimore, Maryland.; ^4^ Department of Radiology, University of Maryland School of Medicine, Baltimore, Maryland.; ^5^ Walter Reed National Military Medical Center, Bethesda, Maryland.

**Keywords:** ASIA Impairment Scale, decompression, MRI, spinal cord injury, trauma

## Abstract

Although decompressive surgery following traumatic spinal cord injury (TSCI) is recommended, adequate surgical decompression is rarely verified via imaging. We utilized magnetic resonance imaging (MRI) to analyze the rate of spinal cord decompression after surgery. Pre-operative (within 8 h of injury) and post-operative (within 48 h of injury) MRI images of 184 motor complete patients (American Spinal Injury Association Impairment Scale [AIS] grade A = 119, AIS grade B = 65) were reviewed to verify spinal cord decompression. Decompression was defined as the presence of a patent subarachnoid space around a swollen spinal cord. Of the 184 patients, 100 (54.3%) underwent anterior cervical discectomy and fusion (ACDF), and 53 of them also underwent laminectomy. Of the 184 patients, 55 (29.9%) underwent anterior cervical corpectomy and fusion (ACCF), with (26 patients) or without (29 patients) laminectomy. Twenty-nine patients (16%) underwent stand-alone laminectomy. Decompression was verified in 121 patients (66%). The rates of decompression in patients who underwent ACDF and ACCF without laminectomy were 46.8% and 58.6%, respectively. Among these patients, performing a laminectomy increased the rate of decompression (72% and 73.1% of patients, respectively). Twenty-five of 29 (86.2%) patients who underwent a stand-alone laminectomy were found to be successfully decompressed. The rates of decompression among patients who underwent laminectomy at one, two, three, four, or five levels were 58.3%, 68%, 78%, 80%, and 100%, respectively (*p* < 0.001). In multi-variate logistic regression analysis, only laminectomy was significantly associated with successful decompression (odds ratio 4.85; 95% confidence interval 2.2–10.6; *p* < 0.001). In motor complete TSCI patients, performing a laminectomy significantly increased the rate of successful spinal cord decompression, independent of whether anterior surgery was performed.

## Introduction

Investigators have shown that continued spinal cord compression (SCC) following experimental or traumatic spinal cord injury (TSCI) negatively affects neurological outcome, including motor and overall functional status.^1–6^ Magnetic resonance imaging (MRI) and computed tomography (CT) myelogram remain the current standards for demonstrating SCC.^7^ In the most recent studies of the timing of operative intervention in TSCI, candidates for decompressive surgery were selected based on SCC as revealed primarily by T2 weighted image (T2WI) or short tau inversion recovery (STIR) sequences on MRI.^7–10^ Decompressive surgery was carried out in those patients with MRI evidence of continued SCC before or after successful closed reduction in the studies of Vale and colleagues,^10^ Papadopoulos and colleagues,^7^ and Fehlings and colleagues;^8^ Vaccaro and colleagues^9^ also performed randomization after the demonstration of SCC by MRI. Nevertheless, although the role of decompression following TSCI is widely acknowledged, we argue that research into the surgical management of TSCI and its long-term outcome is potentially confounded by lack of post-operative confirmation of actual decompression following operative intervention. Without such studies, the exact definition of “decompression” remains uncertain, and as a result, the most effective techniques for achieving it remain unclear.^7–26^

In a 2007 evidence-based systematic review of traumatic subaxial cervical spine injuries, Dvorak and colleagues^27^ developed an algorithm recommending a stand-alone anterior approach for most compression, burst, and distraction injuries, and a posterior or combined anterior and posterior approach for translation/rotation injuries. Their report does not comment on the relationship between operative approach and the extent of spinal cord decompression. In 2017, Aarabi and colleagues^28^ reported that the extent of decompression as confirmed by post-operative MRI influenced grade conversion in American Spinal Injury Association Impairment Scale (AIS) grade A-C patients. We therefore argue that the most effective strategy for achieving true decompression must follow not only the evidence-based algorithm developed by Dvorak and colleagues,^27^ but also one that increases the likelihood of complete spinal cord decompression. Without such data, ongoing debates regarding the most effective surgical approach to the injured spinal cord in a given setting—whether anterior/posterior/combination—are insufficiently informed. Here, we tested the hypothesis that in motor complete subaxial TSCI patients undergoing decompressive surgery, operative technique determines the extent of spinal cord decompression.

## Methods

In this retrospective analysis of prospectively collected data, the primary outcome was complete spinal cord decompression on post-operative MRI studies, defined as a continuous column of CSF anterior and posterior to the spinal cord on T2WI or STIR sagittal imaging.

### Cohort

**FIG. 1.**
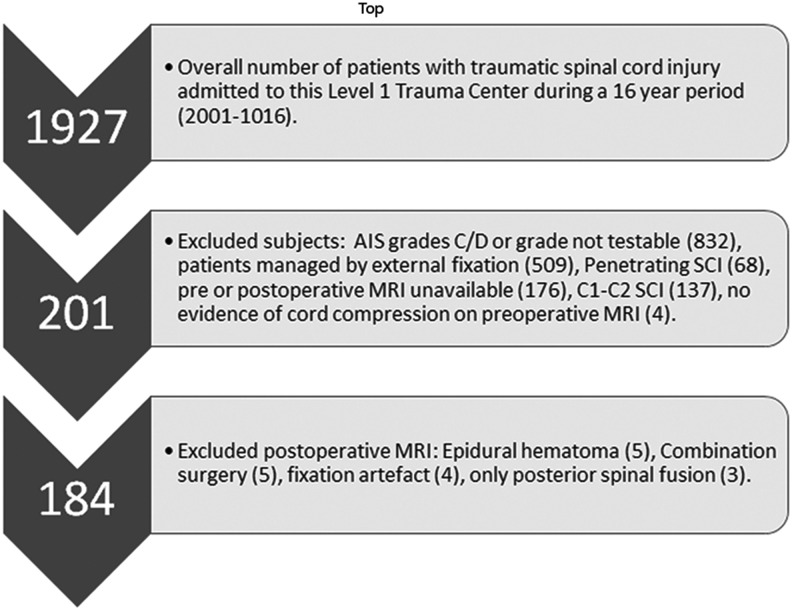
Flow diagram indicating the selection criteria for 184 American Spinal Injury Association Impairment Scale (AIS) grades A and B subaxial cervical spine traumatic spinal cord injury patients who had decompressive surgery following trauma.

From January 2001 through December 2016, over a 16-year period, 1927 patients with blunt subaxial cervical TSCI were admitted to a level I trauma center. Of this number, 184 patients were screened, selected, and included in this study. Patients were age 16 years or older, and based on the International Standards for Neurological and Functional Classification of Spinal Cord Injury (ISNCSCI)^29^ were classified as either AIS grade A or B. Good quality pre- and post-operative CT and MRI studies were available for all patients. The subjects underwent surgery for their injuries and were followed in the intensive or intermediate care unit until discharge to a rehabilitation center or death. Excluded patients included those with AIS grades C and D or non-testable (NT; 832 patients), those with non-operative management of their subaxial cervical spine and spinal cord injuries (509 patients), and those with no or poor imaging studies (197 patients). Also excluded were patients with penetrating (gunshot wound or sharp object) SCI (68 patients; Fig. 1).

Variables including patient demographics, injury mechanism, and severity (Glasgow Coma Scale Score (GCS), Injury Severity Score (ISS), evidence of shock or hypoxemia) were recorded.^30^ We determined American Spinal Injury Association (ASIA) motor score and ASIA Impairment Scale (AIS) grade at admission and following operative intervention.^29,31^ Midsagittal canal diameter and the height of the subaxial cervical spine were measured to control for congenital spinal stenosis.^32,33^ The fracture/dislocation morphology was classified according to the AOSpine subaxial cervical spine injury classification system.^34^ Surgical management of subaxial cervical spine fracture dislocations was compatible with the scheme used in the randomized prospective trial of Vaccaro and colleagues^9^ and the management algorithm of Dvorak and colleagues^27^ We reviewed post-operative CT and MRI studies in order to determine the surgical technique (i.e., stand-alone anterior cervical discectomy and fusion [ACDF], anterior cervical corpectomy and fusion [ACCF], or laminectomy, or in combination). Using the T2WI or STIR sequences, we measured pre-operative and post-operative intramedullary lesion length (IMLL)^28,35^ and determined the state of (obliteration or patency) the subarachnoid space (SAS).^28^ The study started following Investigational Review Board approval of the research proposal.

### Initial rescue, resuscitation, and imaging studies

Patients were initially searched and rescued from the scene of the accident^36^ by emergency medical technicians. Trauma victims were transferred to the Trauma Resuscitation Unit (TRU), back-boarded, and chin-strapped.^28,37,38^ The median time between injury and arrival to TRU for 183 patients was 1.1 h, and the scene time was unknown for one patient. The mean scene time in 140 patients who were directly transferred to the TRU was 1 ± 0.5 h (median 1.0; range 0.2–3.0 h).^28^ In 43 patients who were transferred to the TRU from another facility, the time to arrival was 9.1 ± 10 h (median 5.0; range 3.5–53.5 h). In the TRU, primary and secondary surveys were performed before neurosurgical consultation. Following resuscitation, the patients were sent for CT and MRI of the cervical spine. Pre-operative multi-planar CT of head, chest, abdomen, pelvis, and spine was performed after a mean of 4.1 ± 7.0 h (median 2.1; range 0.5–57.5 h) from injury. The exact delay from injury to CT studies was unknown in one patient. Multi-planar multi-sequence pre-operative MRI images were acquired a mean of 8.4 ± 8.0 h (median 6.0; range 1.7–57.5 h) from injury. MRI was acquired either before or after closed cervical traction reduction in some patients.^28,39^ Post-operative MRI scans were acquired 48.9 ± 30.7 h following injury (median 27 h; range 12–150 h).^28,37^ For one patient, the time interval between injury to pre-operative and post-operative MRI studies was unknown.

### Normal anatomy, injury morphology, and instability

**FIG. 2.**
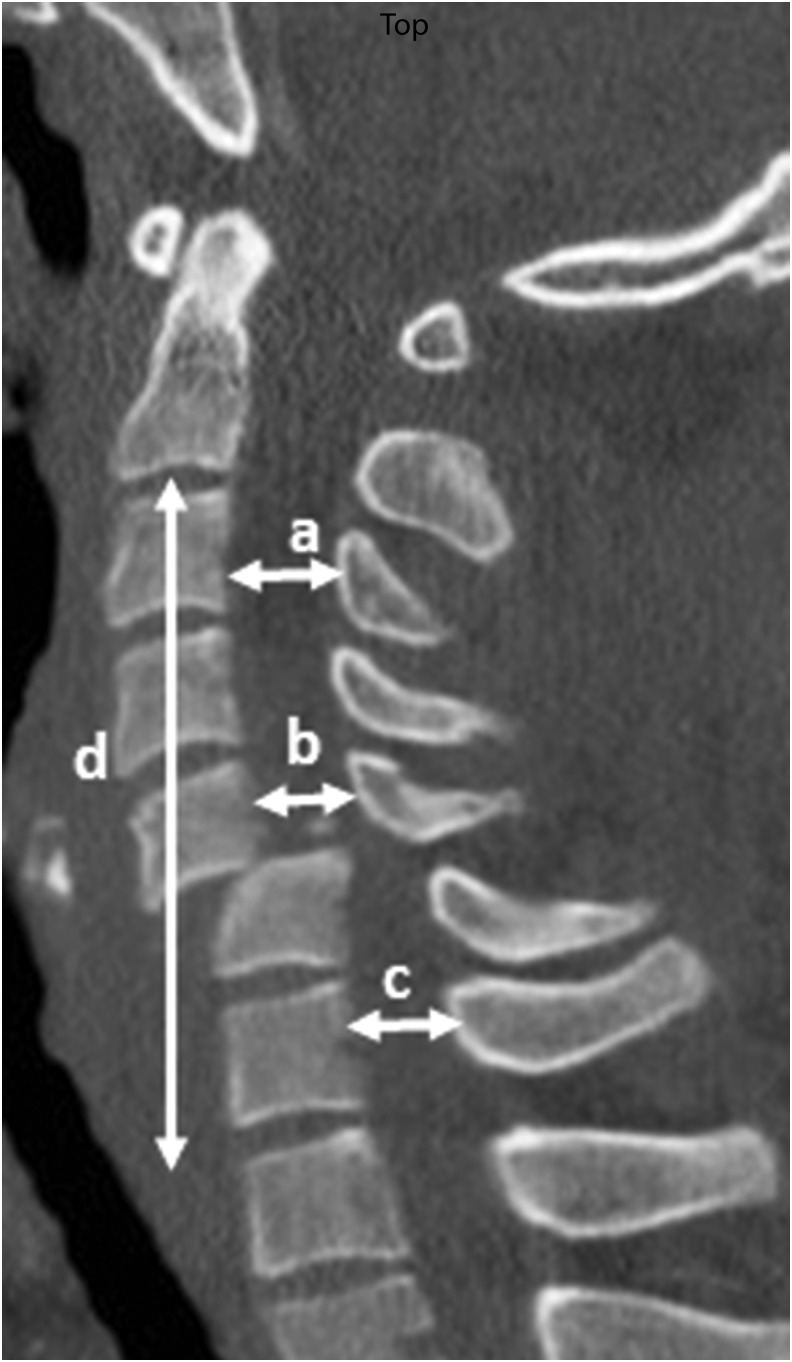
Midsagittal plane of CT scan from subaxial cervical spine indicating methodology of measurements of sagittal diameter at three segmental levels and the subaxial cervical spine height from C2-T1.

**FIG. 3.**
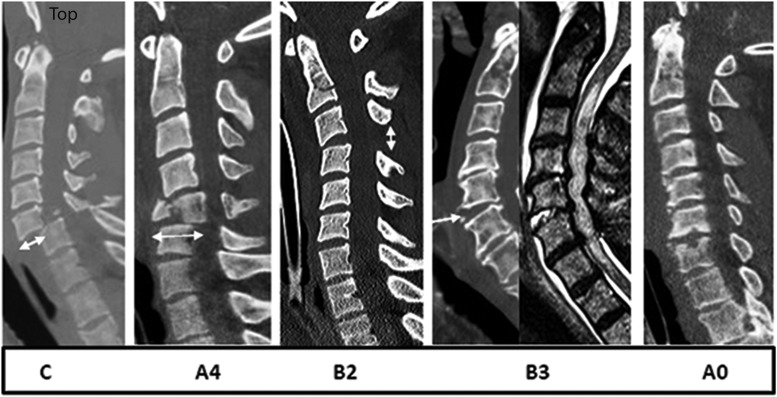
Midsagittal subaxial CT and MRI cuts indicating morphology according to AOSpine Subaxial Cervical Spine Classification System.^34^ C, translation rotation and highly unstable injuries; A4, compression burst fractures; B2, flexion distraction without translation; B3, extension distraction without translation; A0, no imaging evidence of fracture dislocation.

We controlled for congenital spinal stenosis by calculating the mean mid-sagittal A-P diameter of the subaxial cervical spine at three different segmental levels (Fig. 2), applying the methodology recommended by Furlan and colleagues.^33^ Additionally, the height of the subaxial cervical spine was measured from the C2–T1 vertebrae. The AOSpine subaxial cervical spine classification system was used in order to determine injury morphology (Fig. 3).^34^ In this system, compression fractures are classified as types A3 or A4, distraction injuries as types B2 or B3, and injuries associated with translation as type C. To determine fracture/dislocation instability we applied the checklists of White and Panjabi^40^ or Vaccaro and colleagues.^41^

### Intramedullary lesion length and verification of spinal cord decompression

**FIG. 4.**
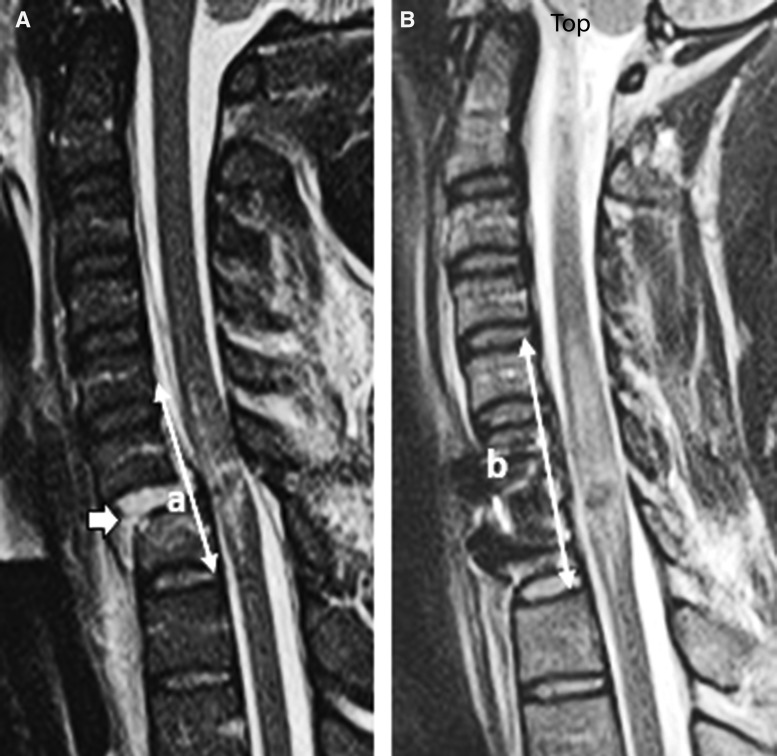
Pre-operative **(A)** and post-operative **(B)** midsagittal magnetic resonance images indicating measured intramedullary lesion length a and b.

Intramedullary lesion length (IMLL) was measured on pre-operative and post-operative MRI (T2WI or STIR) images (Fig. 4).^28,35,37,39^ The mean time from trauma to pre-operative MRI was 8.4 ± 8.0 h (median 6.0; range 1.7–57.5 h). The mean time from injury to post-operative MRI was 48.9 ± 30.7 h (median 37; range 12–150 h).^28,39^ For one patient, the exact time of MRI acquisition following trauma was not recorded. IMLL on pre-operative and post-operative MRI was recorded as the mean of the measured values by four blinded investigators: the principal investigator (B.A.), a trauma neuroradiologist (K.S.), a spine fellowship certified neurosurgeon (K.C.), and a neurotrauma fellowship trained neurosurgeon (G.S.). Decompression was verified by a spine fellowship trained neurosurgeon (C.S.) and a neurotrauma fellowship trained neurosurgeon (G.S.). If there was disagreement between the two raters, the principal investigator (B.A.) served to resolve the discrepancy.

### Surgical intervention and technique

**FIG. 5.**
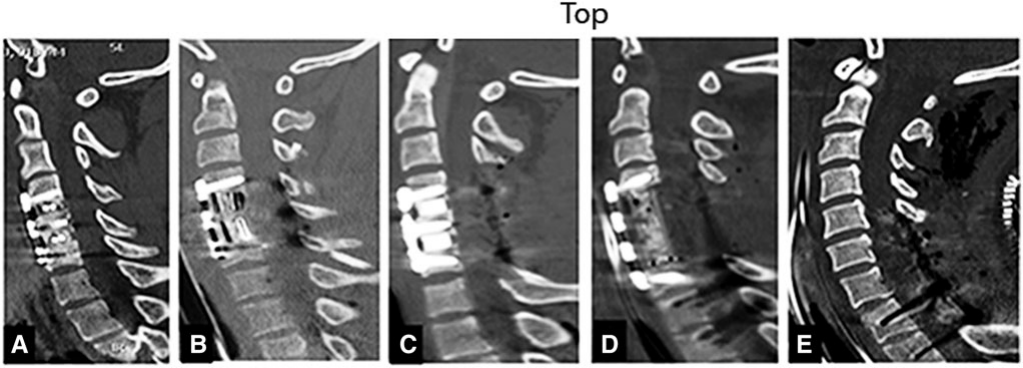
Midsagittal CT scan of cervical spine indicating **(A)** anterior cervical discectomy and fusion (ACDF); **(B)** anterior cervical corpectomy and fusion (ACCF); **(C)** Combined ACDF and three level laminectomy and posterior spinal fusion (PSF); **(D)** ACCF (two levels) and Laminectomy (two levels) and PSF; and **(E)** three levels of laminectomy and PSF.

Decompressive surgery was performed a mean of 21.5 ± 20.8 h (median 14; range 4.0–136.8 h) following trauma by one of 14 board -certified neurosurgeons (five were spine-fellowship trained). When appropriate, certain patients underwent attempted closed reduction by cervical traction prior to operative intervention. Whether or not reduction was achieved, all pre-operative candidates had evidence of spinal cord compression as indicated by deformation and effacement of SAS at the injury epicenter, and either rostrally or caudally to it.^28^ The objective of surgery was anatomical alignment and internal fixation (anterior, posterior, or a combination of anterior and posterior) of the subaxial cervical spine and decompression of the spinal cord.^28^ There was no policy or guidelines for surgical procedures. Each neurosurgeon applied his preferred surgical techniques to obtain decompression and the most secure internal fixation.^28,38^ The five common operative techniques chosen by surgeons were: 1) ACDF (one to three skeletal segments); 2) ACCF (one or two skeletal segments); 3) ACDF in association with laminectomy (one to five levels) with or without posterior spinal fusion; 4) ACCF and laminectomy (one to five levels); and 5) laminectomy (two to five levels) with or without posterior spinal fusion. The surgical techniques used by the 14 neurosurgeons were compatible with the recommended surgical approaches reported previously by Vaccaro and colleagues^9^ and Dvorak and colleagues^27^ (Fig. 5).

### Steroid protocol

From 2001 through 2010 we administered methylprednisolone (30 mg/kg in 15 min within the 1st h and 5.4 mg/kg/h for the next 23 h)^42^ to all patients with TSCI. Infusion started within the first 8 h of trauma. After 2010, this steroid protocol was abandoned at our institution.^43^

### Critical care and hospital length of stay

Following surgery, the patients were kept in the intensive or intermediate care units until discharge or death.^28,37,39^ Fifteen (8.4%) patients died during their acute care in this trauma center. For the 169 patients who were discharged to a rehabilitation center, the mean hospital length of stay was 24.2 ± 8 days (median 19.1; range 3.3–127.5 days). The mean survival for the 15 patients who died was 17.5 ± 15.9 days (median 12.3 days; range 3.3–59.6 days).

### Statistical analysis

Descriptive statistics were calculated. Unpaired *t*-tests and Wilcoxon rank sum tests were used to assess parametrically and non-parametrically distributed variables, respectively. Univariate logistic regression was performed, followed by multi-variable logistic regression to identify variables independently associated with the outcome of successful decompression. Analysis of variance was performed. Variables for the multi-variate regression analysis were chosen after stepwise selection using Bayesian Information Criteria (BIC) to measure the efficiency of the parameterized model in terms of predicting data.^44^ Multiple effect modification terms were assessed, but no evidence of statistical interaction was detected. A final model was chosen based on the model with the smallest BIC value. Regression diagnostics were performed, consisting of Pearson chi-squared goodness-to-fit testing, tests for collinearity and specification, and examination of Pearson residual plots for influential observations. A *p* value of <0.05 was considered statistically significant, and all tests were two-tailed. All analyses were performed in Stata/SE version 15.1 (Stata Corp, College Station, TX) and GraphPad Prism version 7.0d (GraphPad Software, La Jolla, CA).

## Results

We examined 13 variables to assess whether they could in isolation or in regression models influence the achievement of successful decompression of a severely contused and swollen spinal cord across multiple motion segments (Table 1).

**Table 1.** Unadjusted Outcomes, Stratified by Covariates, Including Type of Operation (n = 184)

*Variable*	*Decompressed (%)*	*Not decompressed (%)*	*p value*
Mean age (SD)	45.1 (19.1)	40.3 (18.1)	0.10
Sex (% in each group)			0.55
Male	96 (64.4)	53 (35.6)	
Female	25 (71.4)	10 (15.9)	
Mechanisms of injury (% total in each group)			0.69
Motor vehicle crash	40 (33.1)	29 (46)	
Fall	55 (45.5)	17 (27)	
Sports accident	19 (15.7)	12 (19.1)	
Other	7 (5.8)	5 (7.9)	
Median Injury Severity Score (IQR)	26 (21,35)	29 (25,50)	0.08
Mean ASIA motor score (SD)	15.4 (13.1)	10.1 (8.6)	0.004
ASIA impairment scale (% total in each group)			0.001
A	68 (57.1)	51 (42.9)	
B	53 (81.5)	12 (10.1)	
Mean cervical canal diameter in mm (SD)	12.5 (1.5)	12.3 (1.4)	0.40
Mean cervical vertebral height in mm (SD)	93.6 (7.7)	95.5 (7.2)	0.10
Injury type (on CT, %)			0.68
Translation rotation (C)	42 (61.7)	26 (38.2)	
Burst/Compression fracture (A3 and A4)	28 (68.3)	13 (31.7)	
No fracture/dislocation (A0)	29 (74.4)	10 (25.6)	
Distraction injury (B2 and B3)	16 (61.5)	10 (38.5)	
Other	6 (60)	4 (40)	
Median hours to operation (IQR)	20.9 (20.6)	22.8 (21.5)	0.56
Surgical procedure			0.004
Discectomy	22 (46.8)	25 (53.2)	
Corpectomy	17 (58.6)	12 (41.4)	
Discectomy and laminectomy	38 (71.7)	15 (28.3)	
Corpectomy and laminectomy	19 (73.1)	7 (26.9)	
Laminectomy only	25 (86.2)	4 (13.8)	
Mean pre-operative IMLL in mm (SD)	43.5 (15.3)	55.8 (18)	<0.0001
Mean post-operative IMLL in mm (SD)	62.4 (26)	87.7 (36)	<0.0001
Laminectomy (%)	39 (51.3)	37 (48.7)	<0.001

SD, standard deviation; IQR, interquartile range; CT, computed tomography; IMLL, intramedullary lesion length.

### Demographics and injury mechanisms

Although patients who had complete decompression were about 5 years older than patients who were not successfully decompressed, this age difference did not reach statistical significance. Neither was the patient's sex found to influence the success of decompression (*p* = 0.55). Three well-known mechanisms of injury—motor vehicle accidents, mechanical falls, and sport injuries—did not affect decompression (*p* = 0.69)

### Time interval from injury to admission, imaging studies, and decompression

The mean time interval from injury to admission to the TRU in the decompressed patients was 2.7 ± 4.3 h compared with 3.1 ±6.7 h in patients with unsuccessful decompression (*p* = not significant [NS]). The time interval from injury to CT scan between the two groups (4.1 ± 7.0 and 4.2 ± 6.9 h) did not yield a significant influence on successful decompression. Similarly, the time interval from trauma to pre-operative MRI in the successfully decompressed patients was 8.3 ± 7.7 h, and in the group whose decompression failed it was 8.6 ± 8.7 h (*p* = NS). The time from injury to post-operative MRI in the decompressed group was 49.4 ± 32.6 h, and in the group with incomplete decompression it was 48.2 ±30.0 h (*p* = NS). The median time to surgery in the patients who had successful decompression was 21.0 ± 20.6 h, and in the failed decompression group it was 22.8 ± 21.5 h (*p* = 0.56). In all, the measured time intervals were grossly similar and did not influence the achievement of successful spinal cord decompression as measured on post-operative MRI.

### Evidence of systemic injury severity

At the time of arrival, the mean systolic blood pressure was 119 mm Hg (median 118 mm Hg). Overall, in 17 patients the systolic blood pressure was <90 mm Hg before resuscitation. Systemic oxygenation (SPO_2_) was unknown in 32 patients; however, in 152 patients, the mean SPO_2_ was 97% (median 98%). Median GCS score was 15. GCS score was 3–5 in 10, 6–8 in five, 9–12 in 28, and 13–15 in 141. The mean ISS was 33.2 (median 26; range 1–75). Median ISS (interquartile range) in successfully decompressed patients was 26, and in those with unsuccessful decompression it was 29 (*p* = 0.08).

### ASIA motor score and AIS grade

On the ISNCSCI scale, the mean ASIA motor score in all patients was 13.6 ± 12 (median 10; range 0–50). ASIA motor score was unknown for one patient. ASIA motor score for the patients who had complete spinal cord decompression was 15.4 ± 13.1, and for patients with unsuccessful decompression it was 10.1 ± 8.6. This difference was statistically significant (*p* < 0.004). Additionally, AIS grade A patients were less likely to have successful decompression. The success of decompression in AIS grade A patients was 57.1%, and in AIS grade B it was 81.5% (*p* < 0.001).

### Normal anatomy of cervical spine

Mean midsagittal diameter and the C2–T1 height of the subaxial cervical spine were not significantly different between the two groups (*p* = 0.40 and *p* = 0.10, respectively; Table 1).

### Injury morphology

We compared the AOSpine subaxial cervical spine classification^34^ subtypes between the two groups, but found no statistically significant difference between the morphology patterns of successfully and unsuccessfully decompressed patients (Table 1; *p* = 0.68).

### Surgical technique

To better clarify the operative approaches chosen by the 14 neurosurgeons, we cross-tabulated morphology (Fig. 3) and surgical technique (Fig. 5). Most of the 68 patients with translation rotation injuries (e.g., facet locks,45 AOSpine type C^41^) were managed by either ACDF alone or ACDF with laminectomy and posterior spinal fusion (PSF; 78%). Injuries to the vertebral body such as burst or compression fractures^45^ (AOSpine types A3 and A4)^41^ were approached by either ACCF or ACCF and laminectomy and PSF (95.2%). Almost half of the 39 patients with no visible fracture dislocation (AOSpine type A0)^41^ had stand-alone laminectomy and PSF (48.7%) or ACDF with or without laminectomy and PSF (41%). Twenty-six patients had distraction injuries^46^ without translation (AOSpine types B2 or B3).^41^ From this group 84.6% were managed with either ACDF alone or ACDF with laminectomy with or without PSF. These surgical techniques were compatible with the recommendations depicted in the management algorithm of Dvorak and colleague,^27^ and the techniques used by Vale and colleagues^10^ and Vaccaro and colleagues^9^ (Table 2).

**Table 2.** Cross-Tabulation of Injury Morphology and Surgical Technique

*Morphology*	*ACDF*	*ACCF*	*ACDF+Laminectomy*	*ACCF+Laminectomy*	*Laminectomy*	*Total*
Translation rotation (Type C)	25 (36.8)	3 (4.4)	28 (41.2)	9 (13.2)	3 (4.4)	68 (37)
Burst/ compression A3+A4 (Type A)	0 (0)	22 (53.7)	1 (2.4)	17 (41.5)	1 (2.4)	41 (22.3)
No visible injury (A0)	8 (20.5)	3 (7.7)	8 (20.5)	1 (2.6)	19 (48.7)	39 (21.2)
Distraction (Types B2 and B3)	11 (42.3)	0 (0)	11 (42.3)	0 (0)	4 (15.4)	26 (14.1)
Other	3 (30)	1 (10)	3 (30)	1 (10)	2 (20)	10 (5.4)
Total	47 (25.5)	29 (15.8)	51 (27.7)	28 (15.2)	29 (15.8)	184 (100)

ACDF, anterior cervical discectomy and fusion; ACCF, anterior cervical corpectomy and fusion.

**FIG. 6.**
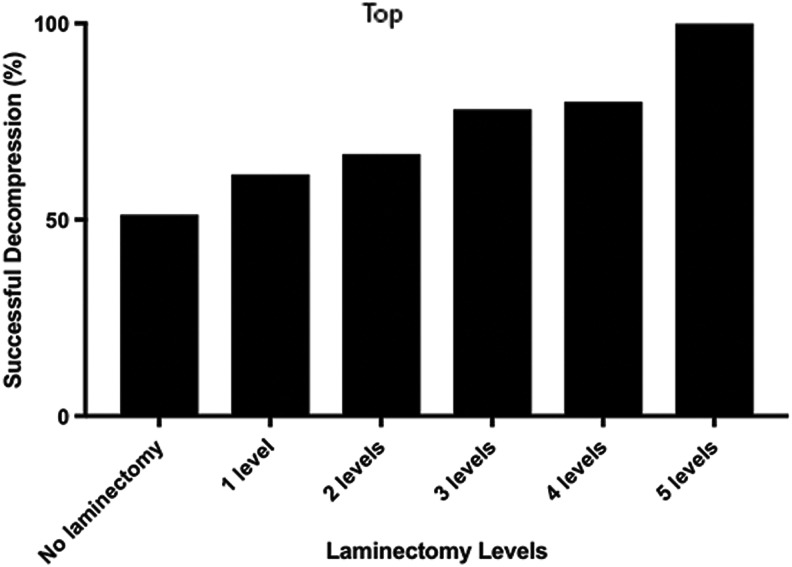
Graph indicating progressively increased chances of success in decompression as the levels of laminectomy increase with surgical technique.

Our study indicated that adding laminectomy to ACDF, ACCF, or stand-alone laminectomy increased the probability of decompression. Additionally, as the number of levels of laminectomy increased from one to five, regardless of the anterior approach chosen, the rate of decompression increased proportionally (Fig. 6).

### ACDF only

**FIG. 7.**
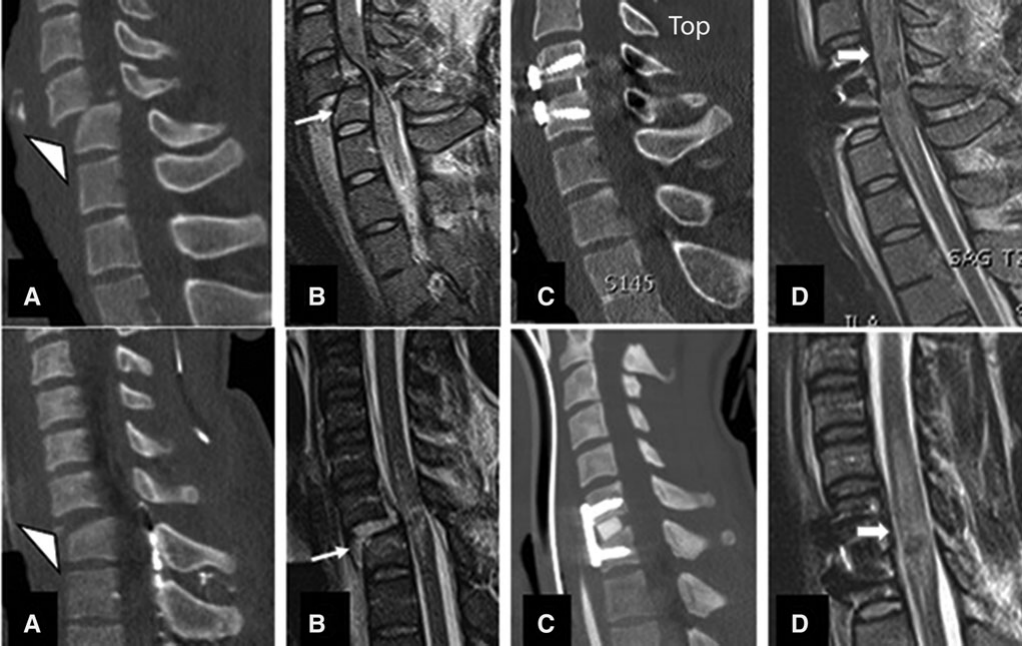
Midsagittal CT and MRI images of two subaxial cervical spine injuries without (upper rows) and with (lower rows) success in decompression. In the upper row a translation rotation injury (**A** and **B**, arrowhead and arrow, respectively) was managed with a single-level ACDF, while the lower row indicates another translation rotation and compression fracture (A and B) managed with a single-level of anterior cervical discectomy and fusion **(C)** and successful decompression (arrow).

Forty-seven of 184 (25.5%) patients had a mean age of 45.0 years and underwent ACDF as their primary surgical approach without laminectomy (Fig. 7). The mean time to surgery was 17.3 h following trauma. In 35 patients, only one skeletal segment was fused. Two-level ACDF was undertaken in nine patients and three-level surgery was employed in three patients. The injury morphology in 36 of 47 (77%) patients was either translation rotation or distraction. The mean ASIA motor score in these patients was 14.7 and AIS grade was A in 33 (70%). IMLL was 45.0 millimeters in the pre-operative MRI and 68.7 millimeters in the post-operative MRI (NS, from the entire cohort IMLL measurements). Only 22 of 47 patients (46.8%) in this group of patients had successful decompression of the spinal cord. Five patients (10.6%) died in this series. The mean hospital length of stay was 23.2 days.

### Corpectomy (ACCF) only

**FIG. 8.**
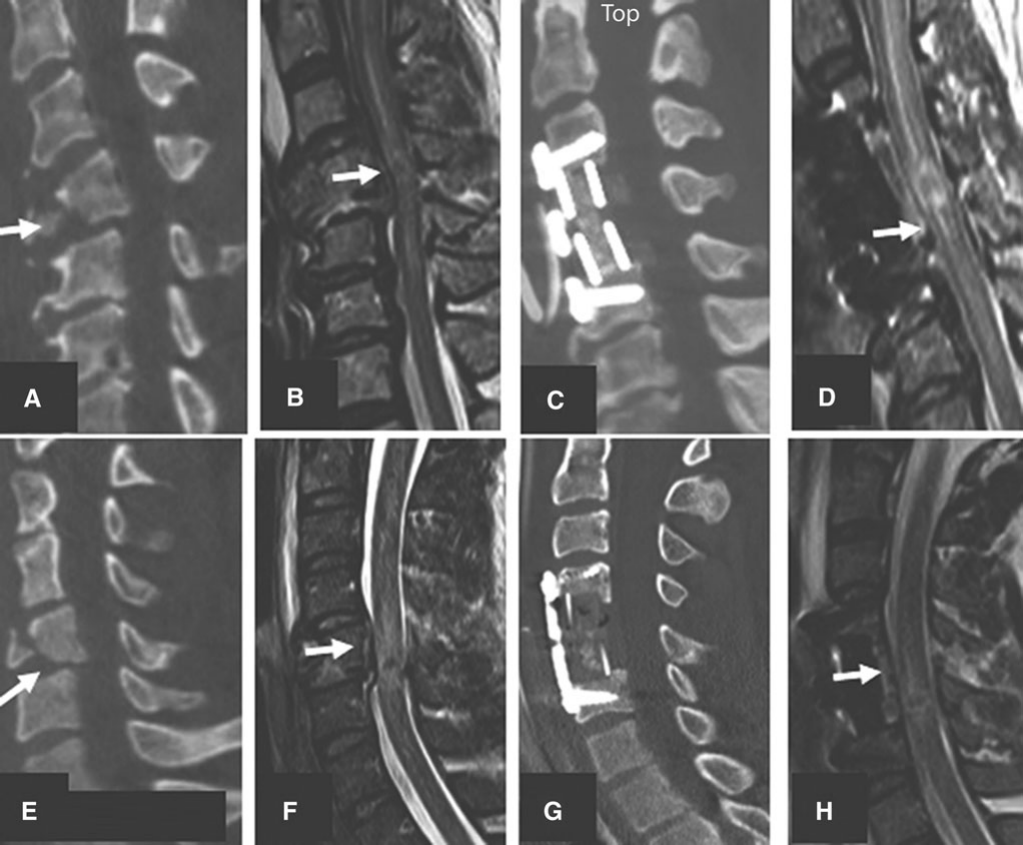
Midsagittal CT and MRI of one level ACCF with and without success in decompression. **(A-D)** indicate a teardrop fracture (A and B arrows) indicating compressed spinal cord at C4 and beyond. (C) and (D) show one level corpectomy with no evidence of decompression (D). **(E)** and **(F)** indicate a C5 tear drop fracture managed with corpectomy and successful decompression of spinal cord (**G** and **H** arrows).

Twenty-nine of 184 (15.7%) patients with a mean age of 29.3 years had corpectomy at one level (28 patients) or two levels (one patient) without laminectomy (Fig. 8). The mean time to surgery following injury was 24.8 h. Mean ASIA motor score in this group was 17.8. The AIS grade was A in 15 (50.7%) and B in 14 patients. Twenty-two of 29 (75.6%) patients had burst or compression fractures (A3 or A4). IMLL was 45.8 millimeters in the pre-operative MRI and 67.9 millimeters in the post-operative MRI. The hospital length of stay in this group was 20.3 days, and two patients died. Seventeen of 29 (58.6%) patients had complete spinal cord decompression (Fig. 8).

### ACDF followed by laminectomy

**FIG. 9.**
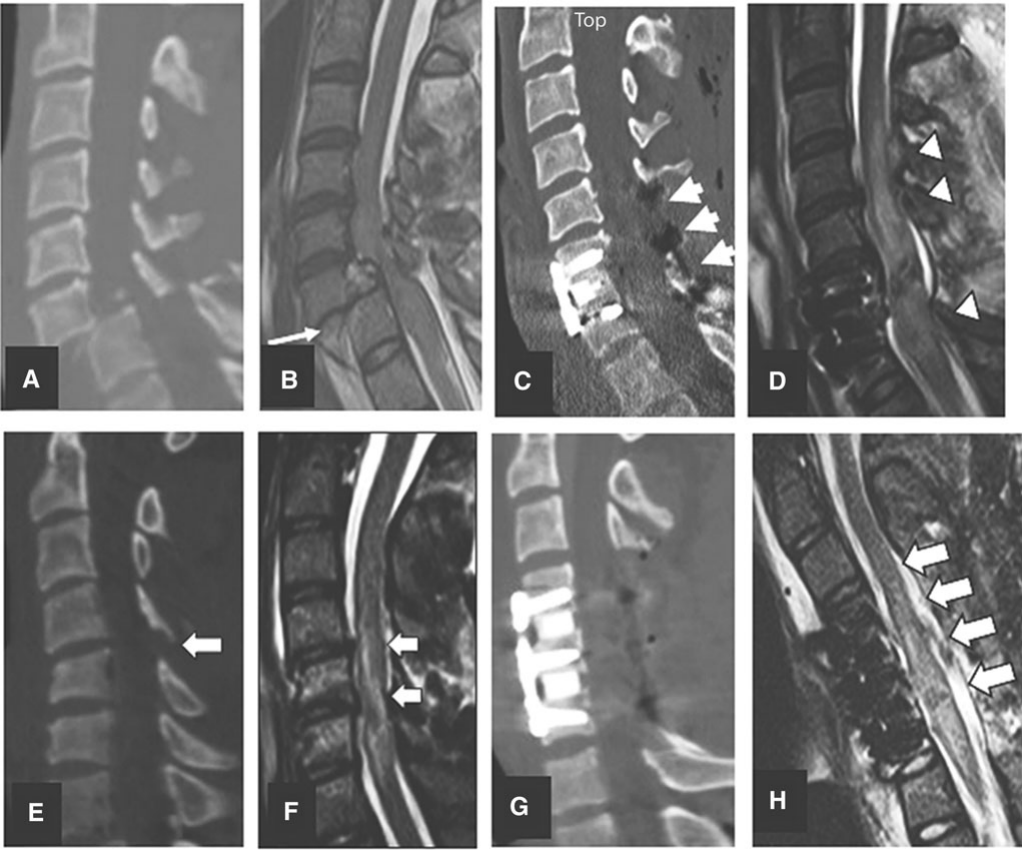
Midsagittal CT and MRI images belonging to two different patients managed by anterior cervical discectomy and fusion (ACDF) and laminectomy. Upper row indicates a C6/C7 translation rotation injury (**A** and **B**) with evidence of spinal cord compression. **(C)** and **(D)** show one level ACDF and two levels of laminectomy with inadequate decompression of the spinal cord at C4 and C5 and C7. **(E-H)** indicate a flexion compression injury and evidence of spinal cord compression at C5 and C6 segments. The patient had ACDF at C5/6 and C6/7 with three levels of laminectomy and posterior spinal fusion (**G** and **H**) with adequate decompression of the spinal cord (arrows).

Fifty-three of 184 (28.8%) patients with a mean age of 47.1 years underwent ACDF followed by laminectomy (Fig. 9). The mean ASIA motor score was 11.9, and the mean time to surgery was 20.1 h following trauma. The injury morphology in this group of patients was translation rotation in 30 and distraction in 11. Of the 53 patients, 34 (53%) underwent one-level ACDF, 13 underwent two-level, five underwent three-level, and one patient underwent four-level ACDF. Laminectomy was one-level in eight, two-level in 15, three-level in 15, four-level in 11, and five-level in four patients. Thirty-eight of 53 (71.7%) patients had complete spinal cord decompression. IMLL was 47.2 and 73.4 millimeters in the pre-operative and post-operative MRI studies, respectively. Six patients (11.3%) died in this series. The mean hospital length of stay was 28 days.

### ACCF followed by laminectomy

**FIG. 10.**
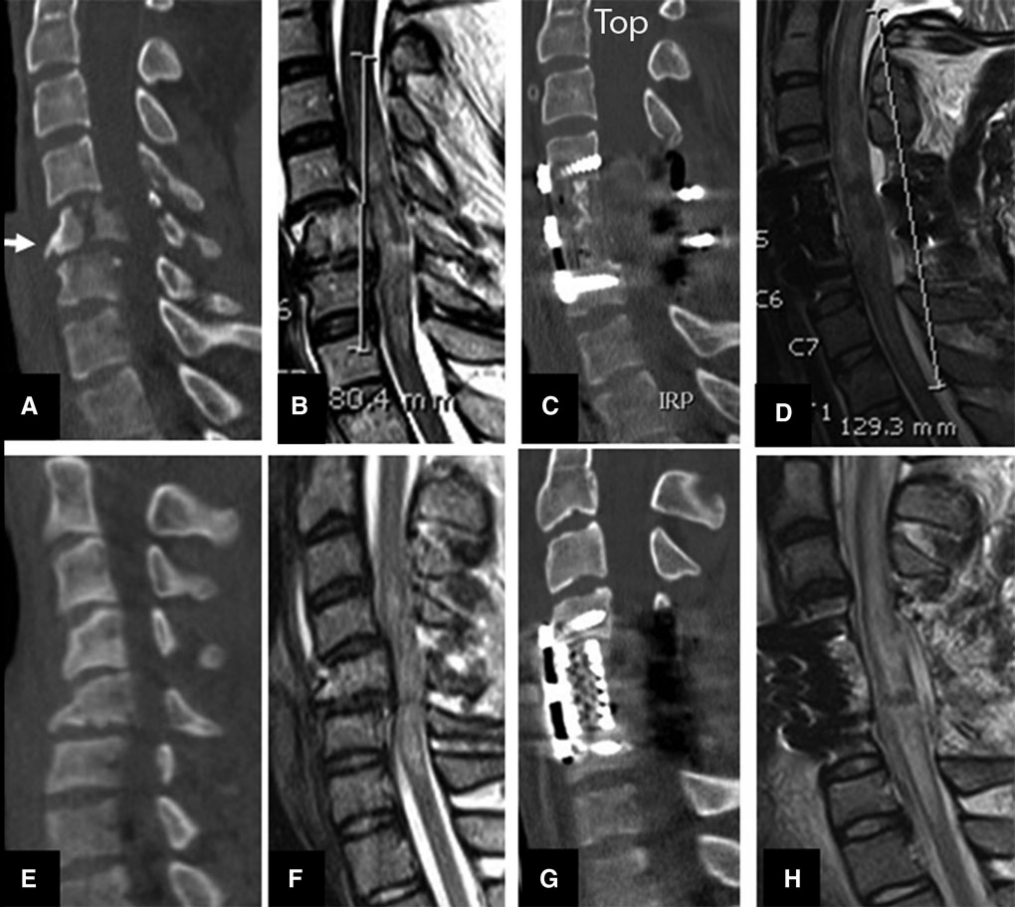
Midsagittal CT and MRI images indicating wo vertical compression and teardrop fractures managed by corpectomy and laminectomy. Upper row plates **(A-D)** indicate C5 corpectomy and three-level laminectomies and posterior spinal fusion (PSF) with inadequate decompression at C3 and C7. Lower row **(E-H)** plates showing C5 corpectomy and three-level laminectomy and PSF with full decompression of the spinal cord.

Twenty-six patients underwent corpectomy with one to five levels of laminectomy (Fig. 10). The mean time to surgery following trauma was 19.7 h in this cohort. Laminectomy was one-level in five patients, two-level in nine, three-level in nine, four-level in two, and five-level in one. The mean age of this group was 31.9 years. SCI was complete in 16 patients. These patients had a mean AISA motor score of 12. The injury morphology was burst/compression or translation rotation in 65.4%. Nineteen patients (73%) in this group had complete decompression of SCI following surgery. Mean IMLL was 54.3 and 80.6 millimeters in the pre-operative and post-operative MRI studies, respectively. None of these 26 patients died. The mean hospital length of stay was 22.9 days.

### Laminectomy only

**FIG. 11.**
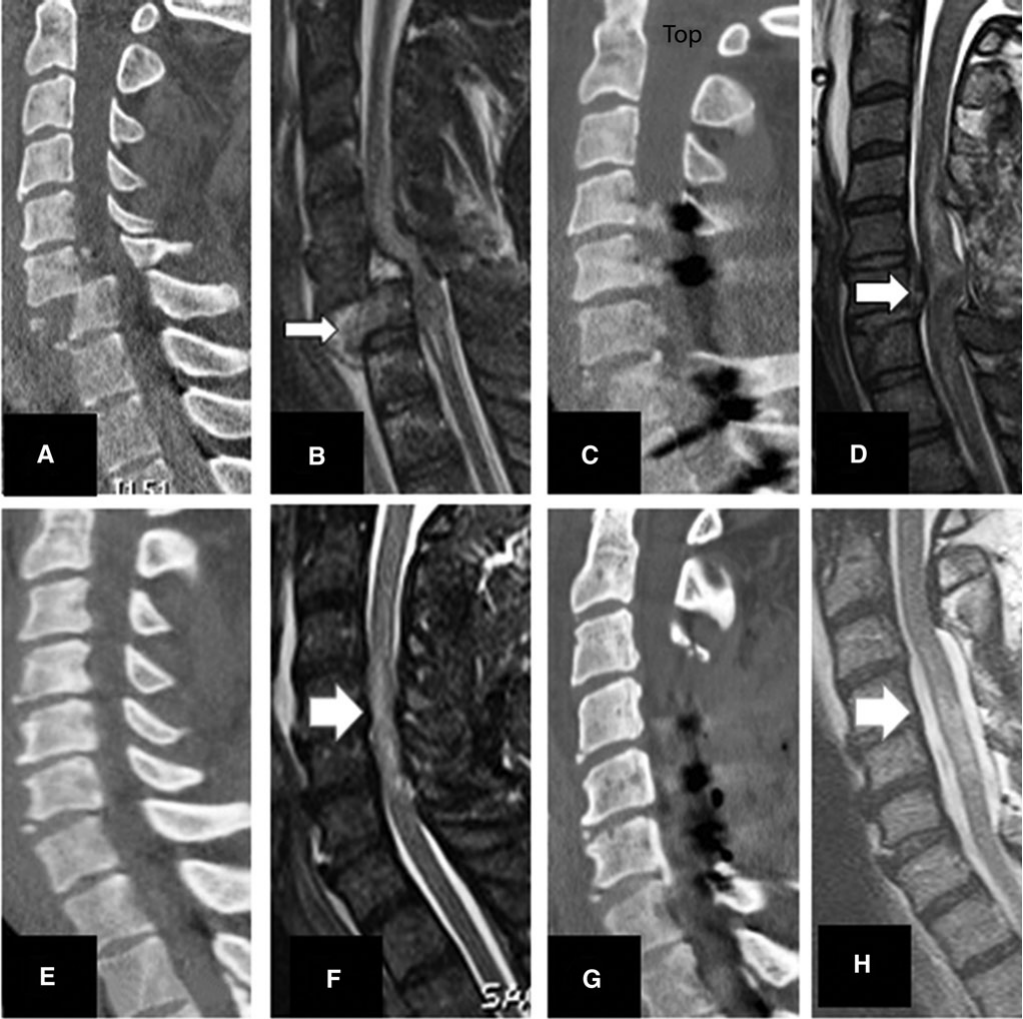
Midsagittal views of two motor complete traumatic spinal cord injury patients with translation rotation **(A-D)** and no evidence of subaxial cervical spine fracture dislocations **(E-H)**. Inadequate laminectomy and posterior spinal fusion (PSF) in the first patient did not decompress the cord adequately (at C7, plate D). Images in the lower row indicate that 4 level laminectomies and PSF completely decompressed the spinal cord.

Twenty-nine motor complete patients underwent laminectomies between two- and five-levels (Fig. 11). The mean time to surgery following trauma was 28.2 h. Laminectomy was two-levels in one patient, three-level in eight patients, four-level in seven patients, and five-level in eight patients. Nineteen of 29 (65.5%) patients in this series had no visible fracture dislocations. AIS grade was almost equally divided between the A (15 patients) and B (14 patients) groups. The mean ASIA motor score was 12. The measured IMLL was 49 and 65.3 millimeters in the pre-operative and post-operative MRI studies, respectively. Two patients died in this series. The mean length of hospital stay was 23.8 days. Twenty-five of 29 patients (86.2%) in this cohort had complete spinal cord decompression. Laminectomy in 22 patients was in conjunction with internal fixation. Seven of 29 patients with stand-alone laminectomy did not have posterior spinal fusion. Morphology in all these seven patients was A0 with no evidence of fracture dislocations. Laminectomy was at two levels in two, three levels in three, and four levels in two patients. Decompression was successful in all seven patients.

### Intramedullary lesion length

Patients with shorter measured IMLL on the pre-operative or post-operative MRI imaging studies were significantly more likely to be completely decompressed (*p* < 0.0001; Table 1).

### Analysis of all five surgical techniques together

Comparison of all of the above-mentioned surgical techniques showed that those employing stand-alone laminectomy or laminectomy with or without PSF as a supplement to an anterior approach were more successful in achieving decompression of the spinal cord (*p* < 0.004; Table 1).

### Multi-variate regression analysis

Analysis of all the co-variates in multiple logistic regression models indicated that laminectomy was important for achieving successful decompression of the spinal cord (Table 3).

**Table 3.** Multivariate Logistic Regression of Covariates (n = 184)

*Variable*	*Unadjusted odds ratio*	*95% CI*	*p value*	*Adjusted odds ratio*	*95% CI*	*p value*
Age	1.01	0.99–1.03	0.10	1.01	0.98–1.03	0.12
Sex	0.72	0.32,1.60	0.42	0.80	0.30–2.13	0.66
ISS	0.99	0.97–1.001	0.07	0.99	0.97–1.02	0.65
Pre-operative IMLL (mm)	0.96	0.94,0.98	< 0.001	0.98	0.96–1.01	0.18
Post-operative IMLL (mm)	0.97	0.96,0.98	< 0.001	0.98	0.96–1.00	0.051
Injury type (on CT, %)						
Fracture/dislocation	1.61	0.99–2.6	0.055	Ref.	Ref.	Ref.
Compression fracture	1.33	0.58–3.03	0.49	1.48	0.54–4.03	0.44
No fracture/dislocation	1.79	0.75–4.28	0.19	0.83	0.28–2.4	0.73
Distraction injury	0.99	0.39–2.5	0.98	0.37	0.11–1.29	0.12
Other	0.93	0.24–3.6	0.92	0.63	0.13–3.01	0.57
Laminectomy	2.99	1.6–5.61	0.001	4.85	2.2–10.6	< 0.001

CI, confidence interval; ISS, Injury Severity Score; IMLL, intramedullary lesion length.

## Discussion

Evidence from this clinical investigation indicates that in motor complete cervical TSCI patients, laminectomy increases the likelihood of complete decompression of the spinal cord. Notably, the rate of successful decompression increases with an increasing number of levels chosen for laminectomy.

**FIG. 12.**
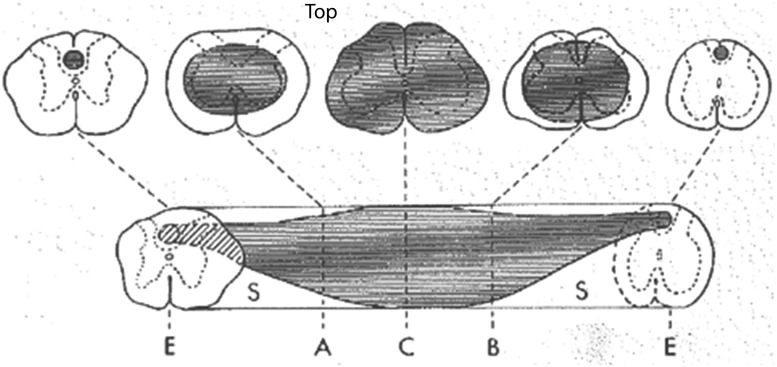
Pre-clinical rodent model of experimental traumatic spinal cord injury indicating an ischemic necrotic spinal cord injury with rostral and caudal expansion over time. From Balentine,^56^ with permission.

Pre-clinical studies have indicated that TSCI leads to capillary disruption at the injury epicenter^47–49^ resulting in the extravasation of blood products within the central gray matter.^48–53^ This ischemia subsequently initiates a number of molecular cascades,^54^ resulting in the upregulation of certain ion channel receptors at the cell membrane,^54,55^ which culminates in the formation of cytotoxic edema and spinal cord swelling potentially well beyond the primary site of initial energy dispersion (Fig. 12).^56^ Spinal cord swelling thus spreads rostrally and caudally from the injury epicenter, leading to the obliteration of the surrounding SAS. The swollen spinal cord becomes compressed against the inelastic dura, and osseous-ligamentous structures of the spinal canal.^28,38,39,57^ *In vivo* imaging studies have confirmed that bleeding at the injury epicenter and spinal cord swelling are clearly visualized on T2WI and SWI sequences.^58–61^ In a rodent model of SCI, Bilgen and colleagues^62^ reported MRI spinal cord swelling within 10 min of trauma followed by the secondary spread of edema beyond the site of impact on subsequently acquired imaging studies. In humans, Le and colleagues and Aarabi and colleagues reported an IMLL expansion rate of nearly 900 μmeters/h when comparing pre-operative with post-operative MRI studies of patients with motor complete subaxial TSCI.^38,39^

The findings of the present study confirm the phenomenon of secondary spinal cord swelling over time following the initial trauma. IMLL, an indicator of longitudinal spinal cord swelling, measured an average of 47.7 mm on the initial pre-operative MRI studies of the patients in this cohort (mean time from injury, 8.4 h). Comparatively, the mean IMLL was 71.1 mm on the post-operative MRI studies (mean time from injury, 48.9 h), resulting in an overall 49% increase in lesion length. While the mean height of C2-T1 osseous-ligamentous motion segments was 94.3 mm (standard deviation 7.5 mm), in 38 of 184 (20.6%) patients the IMLL ranged from 94.3 to 191.8 mm. One important implication of these findings is that the unchecked progression of secondary spinal cord swelling may extend beyond the subaxial cervical spine proper, toward the brainstem rostrally and caudally below T1, thereby increasing the morbidity and mortality of these patients.

Planning an appropriate operative intervention, which increases the likelihood of complete decompression of the spinal cord, becomes even more significant since decompression may significantly affect AIS grade conversion. In a recent investigation of 100 AIS grade A, B, and C patients with subaxial TSCI, a connection was found between the extent of spinal cord decompression and upward AIS grade conversion during a 6-month follow-up period.^28^ Upward AIS grade conversion was 58.9% in 73 patients with adequate decompression versus 18.5% in patients without adequate decompression (*p* < 0.001). The present study indicates that laminectomy increases the probability of complete decompression (odds ratio 4.85; 95% CI 2.2–10.6; *p* < 0.001).

Recent therapeutic trials confirm the continued importance of present research on the combined effects of the timing of operative intervention and extent of surgical decompression of the traumatically injured cervical spinal cord.^7,8,11,12,15,20,23–25,63–65^ With this in mind, we argue that data collection, analysis, and reporting of results are potentially confounded in trials that neither follow a standardized algorithm for surgical intervention nor attend to the issue of spinal decompression and its post-operative confirmation.^66–68^

According to the most currently accepted management algorithms for managing subaxial TSCI, the presence of SCC at the time of initial radiographic assessment does factor into the decision of operative versus non-operative management. However, the choice of the operative technique itself (whether anterior or posterior or a combination and the rostral-caudal extent of intervention) has been primarily guided to date by the goal of correcting structural instability and misalignment. While our experience largely supports the evidence-based paradigm of Dvorak and colleagues,^27^ our research suggests that it may be necessary to supplement this currently accepted algorithm in order to more adequately address the relationship between the chosen operative approach, the extent of spinal cord decompression, and the re-establishment of structural stability and alignment. Nevertheless, as the preceding discussion shows, the time-dependent process of secondary spinal cord swelling has far-reaching consequences. As a result, surgical strategies must anticipate this phenomenon while addressing the issue of alignment/stability. The most effective strategy must not only follow the evidence-based algorithm of Dvorak and colleagues.^27^ we suggest, but also assure complete spinal cord decompression. With regard to the role of MRI, we suggest that the detection of SCC on admission studies should influence not only the decision to operate but also, in conjunction with admission neurologic status (i.e., AIS grade), the choice of a specific surgical strategy. In this work, we show that the incorporation of laminectomy into the overall surgical strategy is more likely to yield optimal decompressive outcomes.

**FIG. 13.**
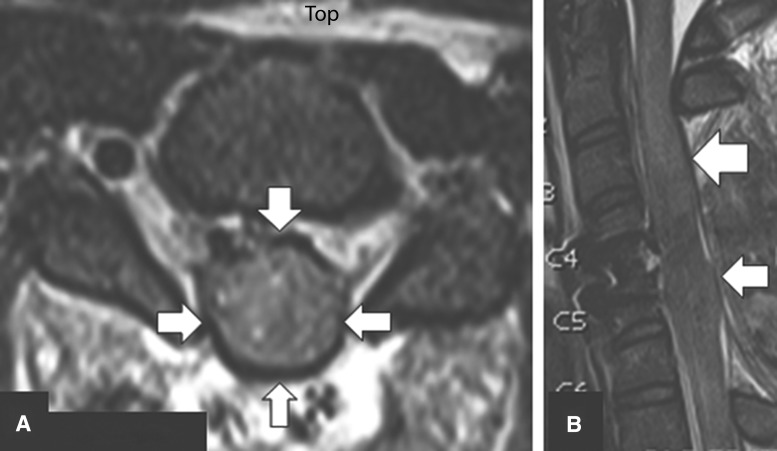
Post-operative axial **(A)** and midsagittal **(B)** MRI images of a motor complete traumatic spinal cord injury indicating severe spinal cord swelling with no visualization of subarachnoid space in the region of laminectomy from C3-C6 (arrows).

Laminectomy, we realize, may also have its limitations for decompressing the injured cervical spinal cord. In our investigation, standard anatomical alignment and decompression re-established a patent SAS in 121 (66%) patients; however, in seven (11%) of 63 patients who had failed decompression, even laminectomy was insufficient for re-establishing patency of the SAS against a non-stretchable dura mater. These patients could have benefited from expansive duraplasty (Fig. 13). Here, there is an analogy to be made with the traumatically injured brain, where expansile duraplasty is almost invariably carried out as part of decompressive craniectomy. Similar to the pathophysiological principles of diffuse traumatic brain injury^69–72^ and the Monro-Kellie Doctrine,^73–75^ it has been shown that increased intraspinal pressure (ISP) results in reduced spinal cord perfusion pressure, as well as changes in the pressure reactivity index (sPRX) and therefore spinal cord autoregulation. In 2015, Phang and colleagues^65^ recorded subdural ISP in patients with severe TSCI and showed that the ISP at the injury epicenter was nearly 10 mm Hg higher than the ISP above or below the level of injury. Of significance was that the subdural ISP was comparable to intraparenchymal ISP at the injury epicenter, consistent with severe spinal cord compression within the region of interest. In a subsequent prospective comparative investigation of 21 patients (11 with laminectomy compared with 10 patients with laminectomy and duraplasty), the investigators^76^ reported improved ISP, spinal cord perfusion pressure (SCPP), and sPRx following expansive duraplasty. This investigation indicates that in a select group of patients, even laminectomy may not sufficiently improve ISP and sPRx, and surgeons may have to perform duraplasty, as is practiced in decompressive craniectomy for diffuse traumatic brain injury.^77–80^ In conclusion, we suggest that within the presently evolving paradigm, further research is required to predict not only which patients will benefit from supplemental or stand-alone laminectomy but also those who will also require duraplasty to adequately decompress the injured cervical spinal cord.

Finally, we acknowledge that this report has inherent weaknesses, as it is not exempt from the potential influence of selection bias. It is a single institution trial, and although we collected the clinical and imaging data prospectively, analysis of the collected data was retrospective in nature.

## Summary

The importance of timely and complete decompression of the contused and compressed spinal cord is gaining wider recognition among neurosurgeons and orthopedic surgeons as evidence increasingly supports its role as a neuroprotective surgical strategy. Nevertheless, the definition of “decompression” still remains heterogeneous and decompression has not been established according to evidence-based guidelines. This study highlights the significance of laminectomy as a supplemental or stand-alone procedure for achieving complete spinal cord decompression.

## References

[1] Fehlings M.G. (2009). The impact of continued cord compression following traumatic spinal cord injury. J. Neurosurg. Spine 11, 568–569 19929359 10.3171/2009.5.SPINE09417

[2] Carlson G.D., Gordon C.D., Oliff H.S., Pillai J.J., and Lamanna J.C. (2003). Sustained spinal cord compression: part I: time-dependent effect on long-term pathophysiology. J. Bone Jt. Surg. Am. 85-A, 86–94 12533577

[3] Kobrine A.I., Evans D.E., and Rizzoli H. (1978). Correlation of spinal cord blood flow and function in experimental compression. Surg. Neurol. 10, 54–59 98855

[4] Kobrine A.I., Evans D.E., and V. R.H. (1979). Experimental acute balloon compression of the spinal cord. Factors affecting disappearance and return of the spinal evoked response. J. Neurosurg. 51, 841–845 115971 10.3171/jns.1979.51.6.0841

[5] Tarlov I.M. and Herz E. (1954). Spinal cord compression studies. IV. Outlook with complete paralysis in man. AMA Arch. Neurol. Psychiatry 72, 43–59 13170854

[6] Tarlov I.M. and Klinger H. (1954). Spinal cord compression studies. II. Time limits for recovery after acute compression in dogs. AMA Arch. Neurol. Psychiatry 71, 271–290 13123590

[7] Papadopoulos S.M., Selden N.R., Quint D.J., Patel N., Gillespie B., and Grube S. (2002). Immediate spinal cord decompression for cervical spinal cord injury: feasibility and outcome. J. Trauma 52, 323–332 11834996 10.1097/00005373-200202000-00019

[8] Fehlings M.G., Vaccaro A., Wilson J.R., Singh A., D, W.C., Harrop J.S., Aarabi B., Shaffrey C., Dvorak M., Fisher C., Arnold P., Massicotte E.M., Lewis S., and Rampersaud R. (2012). Early versus delayed decompression for traumatic cervical spinal cord injury: results of the Surgical Timing in Acute Spinal Cord Injury Study (STASCIS). PloS One 7, e32037 22384132 10.1371/journal.pone.0032037 PMC3285644

[9] Vaccaro A.R., Daugherty R.J., Sheehan T.P., Dante S.J., Cotler J.M., Balderston R.A., Herbison G.J., and Northrup B.E. (1997). Neurologic outcome of early versus late surgery for cervical spinal cord injury. Spine 22, 2609–2613 9399445 10.1097/00007632-199711150-00006

[10] Vale F.L., Burns J., Jackson A.B., and Hadley M.N. (1997). Combined medical and surgical treatment after acute spinal cord injury: results of a prospective pilot study to assess the merits of aggressive medical resuscitation and blood pressure management. J. Neurosurg. 87, 239–246 9254087 10.3171/jns.1997.87.2.0239

[11] Dvorak M.F., Noonan V.K., Fallah N., Fisher C.G., Finkelstein J., Kwon B.K., Rivers C.S., Ahn H., Paquet J., Tsai E.C., Townson A., Attabib N., Bailey C.S., Christie S.D., Drew B., Fourney D.R., Fox R., Hurlbert R.J., Johnson M.G., Linassi A.G., Parent S., and Fehlings M.G. (2015). The influence of time from injury to surgery on motor recovery and length of hospital stay in acute traumatic spinal cord injury: an observational Canadian cohort study. J. Neurotrauma 32, 645–654 25333195 10.1089/neu.2014.3632 PMC4410758

[12] Feng G., Hong Y., Li L., Liu H., Pei F., Song Y., Huang F., Tu C., Li T., Gong Q., Liu L., Zeng J., Kong Q., and Gupte M. (2012). Anterior decompression and nonstructural bone grafting and posterior fixation for cervical facet dislocation with traumatic disc herniation. Spine 37, 2082–2088 22614801 10.1097/BRS.0b013e31825ee846

[13] Krengel W.F. 3rd, Anderson P.A., and Henley M.B. (1993). Early stabilization and decompression for incomplete paraplegia due to a thoracic-level spinal cord injury. Spine 18, 2080–2087 8272964 10.1097/00007632-199310001-00027

[14] La Rosa G., Conti A., Cardali S., Cacciola F., and Tomasello F. (2004). Does early decompression improve neurological outcome of spinal cord injured patients? Appraisal of the literature using a meta-analytical approach. Spinal Cord 42, 503–512 15237284 10.1038/sj.sc.3101627

[15] Lenehan B., Fisher C.G., Vaccaro A., Fehlings M., Aarabi B., and Dvorak M.F. (2010). The urgency of surgical decompression in acute central cord injuries with spondylosis and without instability. Spine 35 (21 Suppl), S180–S186 10.1097/BRS.0b013e3181f32a44 20881460

[16] Levi L., Wolf A., Rigamonti D., Ragheb J., Mirvis S., and Robinson W.L. (1991). Anterior decompression in cervical spine trauma: does the timing of surgery affect the outcome? Neurosurgery 29, 216–222 1886659

[17] Liu Y., Shi C.G., Wang X.W., Chen H.J., Wang C., Cao P., Gao R., Ren X.J., Luo Z.J., Wang B., Xu J.G., Tian J.W., and Yuan W. (2015). Timing of surgical decompression for traumatic cervical spinal cord injury. Int. Orthop. 39, 2457–2463 25576248 10.1007/s00264-014-2652-z

[18] McAfee P.C., Bohlman H.H., Ducker T.B., Zeidman S.M., and Goldstein J.A. (1995). One-stage anterior cervical decompression and posterior stabilization. A study of one hundred patients with a minimum of two years of follow-up. J. Bone Joint Surg. Am. 77, 1791–1800 8550645 10.2106/00004623-199512000-00001

[19] Schlegel J., Bayley J., Yuan H., and Fredricksen B. (1996). Timing of surgical decompression and fixation of acute spinal fractures. J. Orthop. Trauma 10, 323–330 8814573 10.1097/00005131-199607000-00006

[20] Umerani M.S., Abbas A., and Sharif S. (2014). Clinical outcome in patients with early versus delayed decompression in cervical spine trauma. Asian Spine J. 8, 427–434 25187859 10.4184/asj.2014.8.4.427 PMC4149985

[21] van Middendorp J.J., Hosman A.J., and Doi S.A. (2013). The effects of the timing of spinal surgery after traumatic spinal cord injury: a systematic review and meta-analysis. J. Neurotrauma 30, 1781–1794 23815524 10.1089/neu.2013.2932

[22] Wang Q., Xiang L., and Liu J. (2013). Re: Feng G, Hong Y, Li L, et al. Anterior decompression and nonstructural bone grafting and posterior fixation for cervical facet dislocation with traumatic disc herniation. Spine (Phila Pa 1976) 38, 967 23660805 10.1097/BRS.0b013e31828fc937

[23] Wilson J.R., Tetreault L.A., Kwon B.K., Arnold P.M., Mroz T.E., Shaffrey C., Harrop J.S., Chapman J.R., Casha S., Skelly A.C., Holmer H.K., Brodt E.D., and Fehlings M.G. (2017). Timing of decompression in patients with acute spinal cord injury: a systematic review. Global Spine J. 7, 95s–115s 29164038 10.1177/2192568217701716 PMC5684838

[24] Cadotte D.W., Singh A., and Fehlings M.G. (2010). The timing of surgical decompression for spinal cord injury. F1000 Med. Rep. 2, 67 21173861 10.3410/M2-67 PMC2990468

[25] Mattiassich G., Gollwitzer M., Gaderer F., Blocher M., Osti M., Lill M., Ortmaier R., Haider T., Hitzl W., Resch H., and Aschauer-Wallner S. (2017). Functional outcomes in individuals undergoing very early (< 5 h) and early (5–24 h) surgical decompression in traumatic cervical spinal cord injury: analysis of neurological improvement from the Austrian Spinal Cord Injury Study. J. Neurotrauma 34, 3362–3371 28683592 10.1089/neu.2017.5132

[26] El Tecle N.E., Dahdaleh N.S., and Hitchon P.W. (2016). Timing of surgery in spinal cord injury. Spine 41, E995–E1004 26909843 10.1097/BRS.0000000000001517

[27] Dvorak M.F., Fisher C.G., Fehlings M.G., Rampersaud Y.R., Oner F.C., Aarabi B., and Vaccaro A.R. (2007). The surgical approach to subaxial cervical spine injuries: an evidence-based algorithm based on the SLIC classification system. Spine 32, 2620–2629 17978665 10.1097/BRS.0b013e318158ce16

[28] Aarabi B., Sansur C.A., Ibrahimi D.M., Simard J.M., Hersh D.S., Le E., Diaz C., Massetti J., and Akhtar-Danesh N. (2017). Intramedullary lesion length on postoperative magnetic resonance imaging is a strong predictor of ASIA Impairment Scale grade conversion following decompressive surgery in cervical spinal cord injury. Neurosurgery 80, 610–620 28362913 10.1093/neuros/nyw053 PMC5748932

[29] American Spinal Injury Association. (1992). International Standards for Neurological and Functional Classification of Spinal cord injury. ASIA/IMSOP: Chicago 10.1038/sj.sc.3100432 9160449

[30] Boakye M., Arrigo R.T., Kalanithi P.S., and Chen Y.R. (2012). Impact of age, injury severity score, and medical comorbidities on early complications after fusion and halo-vest immobilization for C2 fractures in older adults: a propensity score matched retrospective cohort study. Spine 37, 854–859 21971133 10.1097/BRS.0b013e3182377486

[31] Ditunno J.F. Jr., Young W., Donovan W.H., and Creasey G. (1994). The international standards booklet for neurological and functional classification of spinal cord injury. American Spinal Injury Association. Paraplegia 32, 70–80 8015848 10.1038/sc.1994.13

[32] Furlan J.C., Fehlings M.G., Massicotte E.M., Aarabi B., Vaccaro A.R., Bono C.M., Madrazo I., Villanueva C., Grauer J.N., and Mikulis D. (2007). A quantitative and reproducible method to assess cord compression and canal stenosis after cervical spine trauma: a study of interrater and intrarater reliability. Spine 32, 2083–2091 17762809 10.1097/BRS.0b013e318145a91c

[33] Furlan J.C., Kailaya-Vasan A., Aarabi B., and Fehlings M.G. (2011). A novel approach to quantitatively assess posttraumatic cervical spinal canal compromise and spinal cord compression: a multi-center responsiveness study. Spine 36, 784–793 21192294 10.1097/BRS.0b013e3181e7be3a

[34] Vaccaro A.R., Koerner J.D., Radcliff K.E., Oner F.C., Reinhold M., Schnake K.J., Kandziora F., Fehlings M.G., Dvorak M.F., Aarabi B., Rajasekaran S., Schroeder G.D., Kepler C.K., and Vialle L.R. (2015). AOSpine subaxial cervical spine injury classification system. Eur. Spine J. 25, 2173–2184 25716661 10.1007/s00586-015-3831-3

[35] Miyanji F., Furlan J.C., Aarabi B., Arnold P.M., and Fehlings M.G. (2007). Acute cervical traumatic spinal cord injury: MR imaging findings correlated with neurologic outcome–prospective study with 100 consecutive patients. Radiology 243, 820–827 17431129 10.1148/radiol.2433060583

[36] MIEMSS (2015). Maryland Institute for Emergency Medical Services Systems. https://www.nremt.org/rwd/public/states/state-ems-agencies/mol (last accessed 102018)

[37] Aarabi B., Alexander M., Mirvis S.E., Shanmuganathan K., Chesler D., Maulucci C., Iguchi M., Aresco C., and Blacklock T. (2011). Predictors of outcome in acute traumatic central cord syndrome due to spinal stenosis. J. Neurosurgery. Spine 14, 122–130 10.3171/2010.9.SPINE09922 21166485

[38] Le E., Aarabi B., Hersh D.S., Shanmuganathan K., Diaz C., Massetti J., and Akhtar-Danesh N. (2015). Predictors of intramedullary lesion expansion rate on MR images of patients with subaxial spinal cord injury. J. Neurosurgery. Spine, 1–11 10.3171/2014.10.SPINE14576 25746115

[39] Aarabi B., Simard J.M., Kufera J.A., Alexander M., Zacherl K.M., Mirvis S.E., Shanmuganathan K., Schwartzbauer G., Maulucci C.M., Slavin J., Ali K., Massetti J., and Eisenberg H.M. (2012). Intramedullary lesion expansion on magnetic resonance imaging in patients with motor complete cervical spinal cord injury. J. Neurosurg. Spine 17, 243–250 22794535 10.3171/2012.6.SPINE12122 PMC3534760

[40] White A.A. 3rd and Panjabi M.M. (1984). The role of stabilization in the treatment of cervical spine injuries. Spine 9, 512–522 6387954 10.1097/00007632-198407000-00021

[41] Vaccaro A.R., Hulbert R.J., Patel A.A., Fisher C., Dvorak M., Lehman R.A. Jr., Anderson P., Harrop J., Oner F.C., Arnold P., Fehlings M., Hedlund R., Madrazo I., Rechtine G., Aarabi B., andShaninline M.; Spine Trauma Study Group. (2007). The subaxial cervical spine injury classification system: a novel approach to recognize the importance of morphology, neurology, and integrity of the disco-ligamentous complex. Spine 32, 2365–2374 17906580 10.1097/BRS.0b013e3181557b92

[42] Bracken M.B., Shepard M.J., Collins W.F., Holford T.R., Young W., Baskin D.S., Eisenberg H.M., Flamm E., Leo-Summers L., Maroon J., et al. (1990). A randomized, controlled trial of methylprednisolone or naloxone in the treatment of acute spinal-cord injury. Results of the Second National Acute Spinal Cord Injury Study. N. Engl. J. Med. 322, 1405–1411 2278545 10.1056/NEJM199005173222001

[43] Hurlbert R.J., Hadley M.N., Walters B.C., Aarabi B., Dhall S.S., Gelb D.E., Rozzelle C.J., Ryken T.C., and Theodore N. (2013). Pharmacological therapy for acute spinal cord injury. Neurosurgery 72 Suppl 2, 93–105 10.1227/NEU.0b013e31827765c6 23417182

[44] Raftery A.E. (1995). Bayesian model selection in social research. Sociol. Method. 25, 111–163

[45] Harris J.H. Jr., Edeiken-Monroe B., and Kopaniky D.R. (1986). A practical classification of acute cervical spine injuries. Orthop. Clin. North Am. 1, 15–30 3511428

[46] Allen B.L. Jr., Ferguson R.L., Lehmann T.R., and O'Brien R.P. (1982). A mechanistic classification of closed, indirect fractures and dislocations of the lower cervical spine. Spine 7, 1–27 7071658 10.1097/00007632-198200710-00001

[47] Fairholm D.J. and Turnbull I.M. (1971). Microangiographic study of experimental spinal cord injuries. J. Neurosurg. 35, 277–286 22046638 10.3171/jns.1971.35.3.0277

[48] Dohrmann G.J., Wagner F.C. Jr., and Bucy P.C. (1971). The microvasculature in transitory traumatic paraplegia. An electron microscopic study in the monkey. J. Neurosurg. 35, 263–271 22046636 10.3171/jns.1971.35.3.0263

[49] Dohrmann G.J., Wagner F.C. Jr., and Bucy P.C. (1972). Transitory traumatic paraplegia: electron microscopy of early alterations in myelinated nerve fibers. J. Neurosurg. 36, 407–415 4622427 10.3171/jns.1972.36.4.0407

[50] Dolan E.J. and Tator C.H. (1982). The effect of blood transfusion, dopamine, and gamma hydroxybutyrate on posttraumatic ischemia of the spinal cord. J. Neurosurg. 56, 350–358 6799619 10.3171/jns.1982.56.3.0350

[51] Hall E.D. and Wolf D.L. (1986). A pharmacological analysis of the pathophysiological mechanisms of posttraumatic spinal cord ischemia. J. Neurosurg. 64, 951–961 3084721 10.3171/jns.1986.64.6.0951

[52] Locke G.E., Yashon D., Feldman R.A., and Hunt W.E. (1971). Ischemia in primate spinal cord injury. J. Neurosurg. 34, 614–617 4997396 10.3171/jns.1971.34.5.0614

[53] Young W., Flamm E.S., Demopoulos H.B., Tomasula J.J., and DeCrescito V. (1981). Effect of naloxone on posttraumatic ischemia in experimental spinal contusion. J. Neurosurg. 55, 209–219 7252544 10.3171/jns.1981.55.2.0209

[54] Simard J.M., Tsymbalyuk O., Ivanov A., Ivanova S., Bhatta S., Geng Z., Woo S.K., and Gerzanich V. (2007). Endothelial sulfonylurea receptor 1-regulated NC Ca-ATP channels mediate progressive hemorrhagic necrosis following spinal cord injury. J. Clin. Invest. 117, 2105–2113 17657312 10.1172/JCI32041 PMC1924498

[55] Simard J.M., Woo S.K., Norenberg M.D., Tosun C., Chen Z., Ivanova S., Tsymbalyuk O., Bryan J., Landsman D., and Gerzanich V. (2010). Brief suppression of Abcc8 prevents autodestruction of spinal cord after trauma. Sci. Transl. Med. 2, 28ra29 10.1126/scitranslmed.3000522 PMC2903041 20410530

[56] Balentine J.D. (1978). Pathology of experimental spinal cord trauma. I. The necrotic lesion as a function of vascular injury. Lab. Invest. 39, 236–253 713489

[57] Talbott J.F., Whetstone W.D., Readdy W.J., Ferguson A.R., Bresnahan J.C., Saigal R., Hawryluk G.W., Beattie M.S., Mabray M.C., Pan J.Z., Manley G.T., and Dhall S.S. (2015). The Brain and Spinal Injury Center score: a novel, simple, and reproducible method for assessing the severity of acute cervical spinal cord injury with axial T2-weighted MRI findings. J Neurosurg Spine 23, 495–504 26161519 10.3171/2015.1.SPINE141033

[58] Mihai G., Nout Y.S., Tovar C.A., Miller B.A., Schmalbrock P., Bresnahan J.C., and Beattie M.S. (2008). Longitudinal comparison of two severities of unilateral cervical spinal cord injury using magnetic resonance imaging in rats. J. Neurotrauma 25, 1–18 18355154 10.1089/neu.2007.0338

[59] Nout Y.S., Mihai G., Tovar C.A., Schmalbrock P., Bresnahan J.C., and Beattie M.S. (2009). Hypertonic saline attenuates cord swelling and edema in experimental spinal cord injury: a study utilizing magnetic resonance imaging. Crit. Care Med. 37, 2160–2166 19487936 10.1097/CCM.0b013e3181a05d41 PMC2749661

[60] Salegio E.A., Bresnahan J.C., Sparrey C.J., Camisa W., Fischer J., Leasure J., Buckley J., Nout-Lomas Y.S., Rosenzweig E.S., Moseanko R., Strand S., Hawbecker S., Lemoy M.J., Haefeli J., Ma X., Nielson J.L., Edgerton V.R., Ferguson A.R., Tuszynski M.H., and Beattie M.S. (2016). A unilateral cervical spinal cord contusion injury model in non-human primates (Macaca mulatta). J. Neurotrauma 33, 439–459 26788611 10.1089/neu.2015.3956 PMC4799702

[61] Hackney D.B., Ford J.C., Markowitz R.S., Hand C.M., Joseph P.M., and Black P. (1994). Experimental spinal cord injury: MR correlation to intensity of injury. J. Comput. Assist. Tomog. 18, 357–362 10.1097/00004728-199405000-00004 8188899

[62] Bilgen M., Abbe R., Liu S.J., and Narayana P.A. (2000). Spatial and temporal evolution of hemorrhage in the hyperacute phase of experimental spinal cord injury: in vivo magnetic resonance imaging. Magn. Reson. Med. 43, 594–600 10748436 10.1002/(sici)1522-2594(200004)43:4<594::aid-mrm15>3.0.co;2-1

[63] Fehlings M.G., Tetreault L.A., Wilson J.R., Aarabi B., Anderson P., Arnold P.M., Brodke D.S., Burns A.S., Chiba K., Dettori J.R., Furlan J.C., Hawryluk G., Holly L.T., Howley S., Jeji T., Kalsi-Ryan S., Kotter M., Kurpad S., Marino R.J., Martin A.R., Massicotte E., Merli G., Middleton J.W., Nakashima H., Nagoshi N., Palmieri K., Singh A., Skelly A.C., Tsai E.C., Vaccaro A., Yee A., and Harrop J.S. (2017). A clinical practice guideline for the management of patients with acute spinal cord injury and central cord syndrome: recommendations on the timing (</ = 24 hours versus >24 hours) of decompressive surgery. Global Spine J. 7, 195s–202s 29164024 10.1177/2192568217706367 PMC5684850

[64] Hakalo J. and Wronski J. (2004). [Importance of early operative decompression of spinal cord after cervical spine injuries]. Neurol. Neurochir. Pol. 38, 183–188 15354230

[65] Phang I. and Papadopoulos M.C. (2015). Intraspinal pressure monitoring in a patient with spinal cord injury reveals different intradural compartments: Injured Spinal Cord Pressure Evaluation (ISCoPE) Study. Neurocrit. Care 23, 414–418 26136148 10.1007/s12028-015-0153-6

[66] Fehlings M.G., Nakashima H., Nagoshi N., Chow D.S., Grossman R.G., and Kopjar B. (2016). Rationale, design and critical end points for the Riluzole in Acute Spinal Cord Injury Study (RISCIS): a randomized, double-blinded, placebo-controlled parallel multi-center trial. Spinal cord 54, 8–15 26099215 10.1038/sc.2015.95 PMC5399137

[67] Grossman R.G., Fehlings M.G., Frankowski R.F., Burau K.D., Chow D.S., Tator C., Teng A., Toups E.G., Harrop J.S., Aarabi B., Shaffrey C.I., Johnson M.M., Harkema S.J., Boakye M., Guest J.D., and Wilson J.R. (2014). A prospective, multicenter, phase I matched-comparison group trial of safety, pharmacokinetics, and preliminary efficacy of riluzole in patients with traumatic spinal cord injury. J. Neurotrauma 31, 239–255 23859435 10.1089/neu.2013.2969 PMC3904533

[68] Casha S., Zygun D., McGowan M.D., Bains I., Yong V.W., and Hurlbert R.J. (2012). Results of a phase II placebo-controlled randomized trial of minocycline in acute spinal cord injury. Brain 135, 1224–1236 22505632 10.1093/brain/aws072

[69] Donnelly J., Czosnyka M., Adams H., Robba C., Steiner L.A., Cardim D., Cabella B., Liu X., Ercole A., Hutchinson P.J., Menon D.K., Aries M.J.H., and Smielewski P. (2018). Pressure reactivity-based optimal cerebral perfusion pressure in a traumatic brain injury cohort. Acta Neurochir. Suppl. 126, 209–212 29492563 10.1007/978-3-319-65798-1_43

[70] Klein S.P., Bruyninckx D., Callebaut I., and Depreitere B. (2018). Comparison of intracranial pressure and pressure reactivity index obtained through pressure measurements in the ventricle and in the parenchyma during and outside cerebrospinal fluid drainage episodes in a manipulation-free patient setting. Acta Neurochir. Suppl. 126, 287–290 29492576 10.1007/978-3-319-65798-1_56

[71] Zeiler F.A., Donnelly J., Menon D.K., Smielewski P., Hutchinson P.J.A., and Czosnyka M. (2018). A description of a new continuous physiological index in traumatic brain injury using the correlation between pulse amplitude of intracranial pressure and cerebral perfusion pressure. J. Neurotrauma. 2018 Feb 9; Epub ahead of print 10.1089/neu.2017.5241 29212405

[72] Zeiler F.A., Donnelly J., Smielewski P., Menon D.K., Hutchinson P.J., and Czosnyka M. (2018). Critical thresholds of intracranial pressure-derived continuous cerebrovascular reactivity indices for outcome prediction in noncraniectomized patients with traumatic brain injury. J. Neurotrauma. 35, 1107–1115 29241396 10.1089/neu.2017.5472

[73] Huang Y.M. and Davidsson L. (2013). Sagging brain development after lumbar puncture agrees with Monro-Kellie hypothesis. J. Neurology 260, 920–922 10.1007/s00415-012-6811-0 23314405

[74] Karakis I., Nuccio A.H., Amadio J.P., and Fountain A.J. Jr. (2017). The Monro-Kellie doctrine in action: posterior reversible leukoencephalopathy syndrome caused by intracranial hypotension from lumboperitoneal shunt placement. World Neurosurg. 98, 868.e811–868.e815. 10.1016/j.wneu.2016.12.046 28017759

[75] Mokri B. (2001). The Monro-Kellie hypothesis: applications in CSF volume depletion. Neurology 56, 1746–1748 11425944 10.1212/wnl.56.12.1746

[76] Phang I., Werndle M.C., Saadoun S., Varsos G., Czosnyka M., Zoumprouli A., and Papadopoulos M.C. (2015). Expansion duroplasty improves intraspinal pressure, spinal cord perfusion pressure, and vascular pressure reactivity index in patients with traumatic spinal cord injury: Injured Spinal Cord Pressure Evaluation Study. J. Neurotrauma 32, 865–874 25705999 10.1089/neu.2014.3668 PMC4492612

[77] Aarabi B., Hesdorffer D.C., Ahn E.S., Aresco C., Scalea T.M., and Eisenberg H.M. (2006). Outcome following decompressive craniectomy for malignant swelling due to severe head injury. J. Neurosurgery 104, 469–479 10.3171/jns.2006.104.4.469 16619648

[78] Albanese J., Leone M., Alliez J.R., Kaya J.M., Antonini F., Alliez B., and Martin C. (2003). Decompressive craniectomy for severe traumatic brain injury: evaluation of the effects at one year. Crit. care Med. 31, 2535–2538 14530763 10.1097/01.CCM.0000089927.67396.F3

[79] Chibbaro S. and Tacconi L. (2007). Role of decompressive craniectomy in the management of severe head injury with refractory cerebral edema and intractable intracranial pressure. Our experience with 48 cases. Surg. Neurol. 68, 632–638 17765952 10.1016/j.surneu.2006.12.046

[80] Figaji A.A., Fieggen A.G., Argent A.C., Le Roux P.D., and Peter J.C. (2008). Intracranial pressure and cerebral oxygenation changes after decompressive craniectomy in children with severe traumatic brain injury. Acta Neurochir. Suppl. 102, 77–80 19388292 10.1007/978-3-211-85578-2_15

